# Supplementary material for: Mafa-dependent GABAergic activity promotes mouse neonatal apneas
Source: Nat Commun. 2022 Jun 7;13:3284. doi: 10.1038/s41467-022-30825-3 (PMC9174494; doi:10.1038/s41467-022-30825-3)
Supplement: Supplementary file 1 — Supplementary Information [file 41467_2022_30825_MOESM1_ESM.pdf]

Mafa-dependent GABAergic activity promotes mouse neonatal apneas

Supplementary Figures

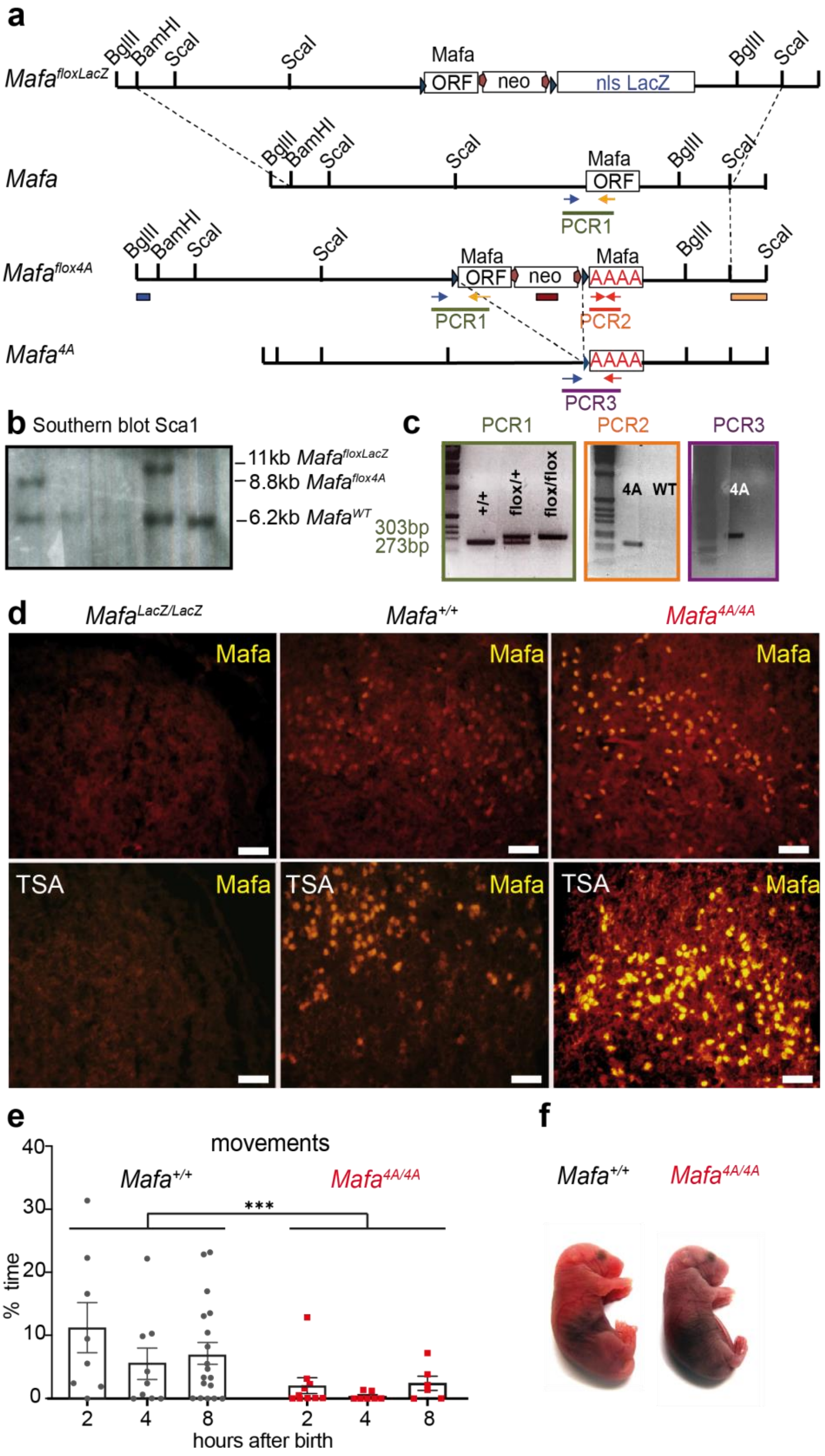

Supplementary Figure 1. Description of the *Mafa* conditional mutants. a In the wild type *Mafa* locus, the open reading frame (ORF) is located on one single exon. To generate conditional alleles, the *Mafa* ORF (with a frt-neo-frt cassette to allow ES cells selection) is flanked by two loxP sites (triangle) and followed by either the reporter gene nuclear LacZ or by the ORF encoding mutated *Mafa-4A*. **b** Recombination was confirmed by Southern blotting of genomic cDNA after digestion by ScaI, hybridized with an external 3'genomic probe (orange box). **c** PCR designed for characterization of the mutated *Mafa-4A* alleles and genotyping: PCR1 flanked the 5' loxP site and allows to distinguish between wild type (273nt) and floxed (303nt) alleles, PCR2 amplifies the mutated 4A allele in *Mafa* ^{flox4A} mutants and PCR3 amplifies the 4A allele after Cre-recombinase deletion of the floxed region in *Mafa*^{4A} mutants. **d** Transverse section at P0 of the dorsal spinal cord immunostained for Mafa without (top panels) or with TSA amplification (bottom panels) confirms the loss of Mafa protein in *Mafa*$^{LacZ/LacZ}$ knock-out mice (n= 3) and the accumulation of Mafa-4A protein in the *Mafa*$^{4A/4A}$ knock-in mice (n=4) compared to Mafa$^{+/+}$ littermates (n=3). **e** *Mafa*$^{4A/4A}$ neonates (red symbols) are hypoactive compared to *Mafa*$^{+/+}$ (grey symbols). Plot showing the movement fraction time (%) during 5 minutes observation periods at postnatal times (2 hours: n=8 control pups and n=10 mutants; 4 hours: n=9 control pups and n=7 mutants; and 8 hours: n=18 control pups and n=6 mutants). For statistical comparison of *Mafa*$^{+/+}$ and *Mafa*$^{4A/4A}$ pups, measurements obtained at all postnatal times have been pooled (two-sided Mann-Whitney test P=0.0005, U=212.5). Plots represent mean+/- sem. **f** *Mafa*$^{4A/4A}$ present with a cyanotic profile compared to their wild type littermates. Scale bars (µm): 50 (**d**).

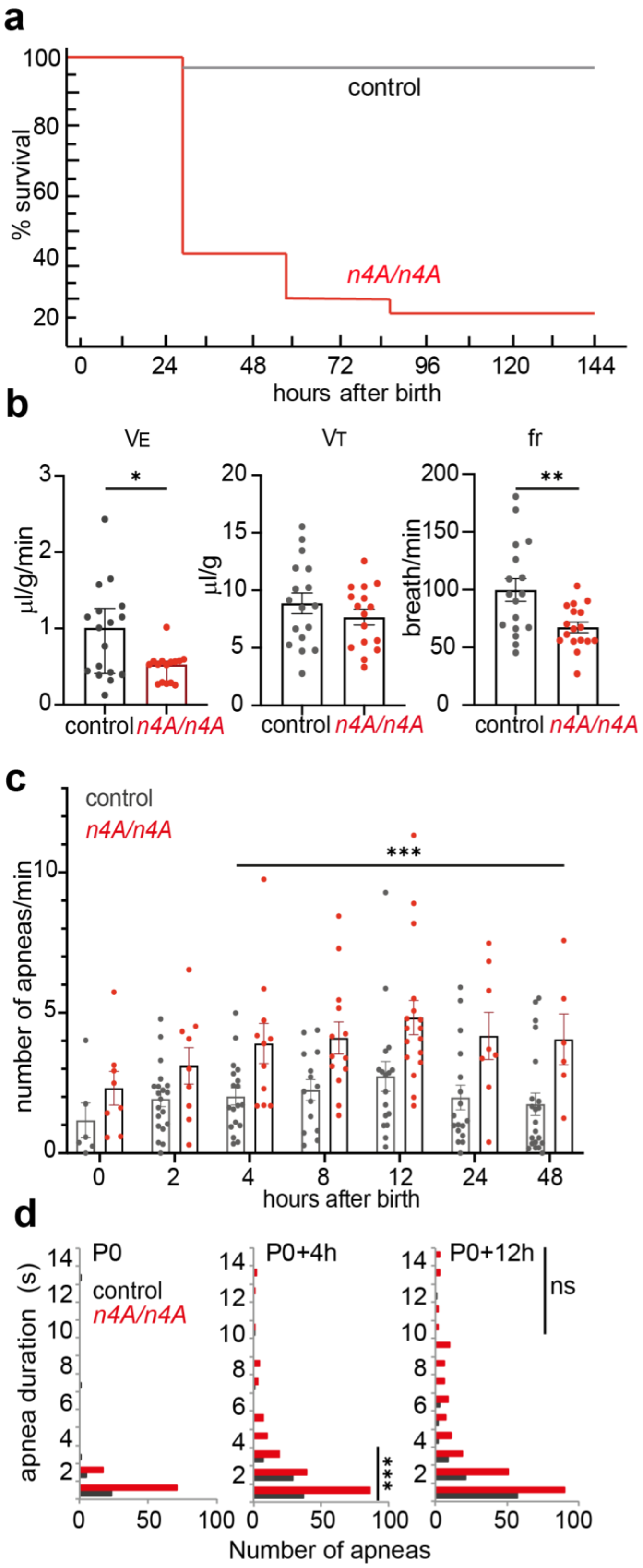
a
100
80
60
40
20
% survival
control
n4A/n4A
0
24
48
72
96
120
144
hours after birth
b
VE
VT
fr
3
2
1
0
µl/g/min
*
20
15
10
5
0
µl/g
200
150
100
50
0
breath/min
**
control
n4A/n4A
c
control
n4A/n4A
***
10
5
0
number of apneas/min
0
2
4
8
12
24
48
hours after birth
d
P0
P0+4h
P0+12h
ns
***
14
12
10
8
6
4
2
apnea duration (s)
0
50
100
Number of apneas

Supplementary Figure 2. Breathing parameters and evolution of the number and duration of apneas in *Mafa*$^{n4A/n4A}$ pups. a Kaplan-Meier survival curve of control (grey, *nestin*$^{+/+}$*;Mafa*$^{flox4A/4A}$, n=23) and *Mafa*$^{n4A/n4A}$ (red, *nestin*$^{Cre/+}$*;Mafa*$^{flox4A/4A}$, n=29). **b** Breathing parameters at P0+12h in control (grey, n=17) and *Mafa*$^{n4A/n4A}$ (red, n=17), minute ventilation V_E (left panel) is decreased in *Mafa*$^{n4A/n4A}$ (two-sided unpaired Student's t-test P=0.01, df=30, t=2.716). This is not due to reduction of the tidal volumes V_T (middle panel, two-sided unpaired Student's t-test P=0.3, df=31, t=1.045) but to a decrease of the breathing frequency fr (two-sided unpaired Student's t-test P=0.0054, df=32, t=2.985). Plots represent mean+/- sem. **c** Histogram of the evolution of the number of apneas per minute between P0 and P2 in control (grey symbols, n=29) and *Mafa*$^{n4A/n4A}$ (red symbols, n=21) mutant pups. In *Mafa*$^{n4A/n4A}$ mutants, the number of apnea is higher than in controls from P0+4h onwards (Two-way Anova P<0.0001, df=1, F=35.67) Plots represent mean+/- sem. **d** Frequency distribution histograms of apnea duration at P0, P0+4h and P0+12h for *Mafa*$^{n4A/n4A}$ (red, n=21) and control (grey, n=29) pups. The about doubled number of short (≤3.5s) apneas at P0+4h (first significant time point) is a much better prodromal sign of the upcoming lethal respiratory distress (two-sided Student's t-test P=0.0257; t=3.464; df=4) than the appearance of long (>10s) apneas. (two-sided Student's t-test P=0.1705; t=1.528; df=7).

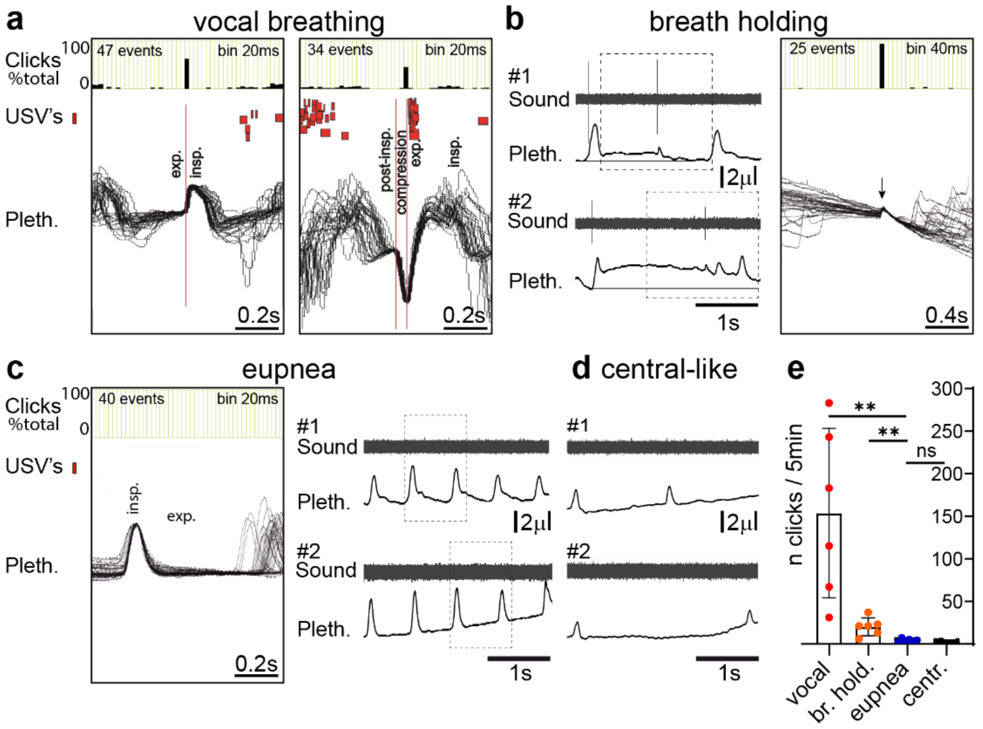

Supplementary Figure 3. Clicks are time-locked to respiratory phase transitions during vocal breathing and breath holding apneas but not during eupnea nor central apneas. a-d Joint audio and plethysmographic analysis of the occurrence of clicks during the respiratory cycle in different breathing modes: vocal (**a**), breath holding apneic (**b**), eupneic (**c**) or central-like apneic (**d**). **a** left panel, time distribution histogram of clicks (top, binning 20ms), occurrence of ultrasonic vocalizations (USV's, red, middle) and superimposed normalized plethysmographic (Pleth, bottom) black traces (n=47 from 4 wildtype P0 pups) synchronized (vertical red line) on the expiratory (exp) / inspiratory (insp., upward deflection) transition; right panel, same with synchronization on the peak compression of the lung that precedes USV's emission (n=34 for 4 wildtype P0 pups). Note that during vocal breathing virtually all clicks are time-locked to inspiratory (left panel) or expiratory (right panel) on-switch. **b** left panel, joint recordings of sound waveform signal (Sound) and plethysmography (Pleth) in two example *Mafa*$^{n4A/n4A}$ mutant P0 pups (#1, #2) during a breath holding apnea. Note that clicks are time-locked to inspiratory on switch and to a small amplitude upward pressure shift on the plethysmographic trace that precedes the first expiratory effort deflating the lung (right, n= 27 superimposed breath holds from 5 *Mafa*$^{n4A/n4A}$ P0 pups); right panel, time distribution histogram of clicks (top, binning 40ms) in register with synchronized and normalized plethysmographic upward pressure shifts (bottom). **c** Absence of clicks during eupneic breathing (40 superimposed respiratory cycles from 3 P0 pups). **d** Absence of clicks during central-like apneas. **e** summary histogram of the frequency (plot represents mean +/- sem) of clicks in different breathing modes, the frequency of clicks is significantly, increased during vocal breathing (two-sided unpaired Student's t-test P=0.041, df=10, t=3.695), breath holding apneas (P=0.0033, df=10, t=3.834), and not significantly changed during central-like apneas (P=0.083, df=1.16, t=1.921) when compared to eupneic breathing.

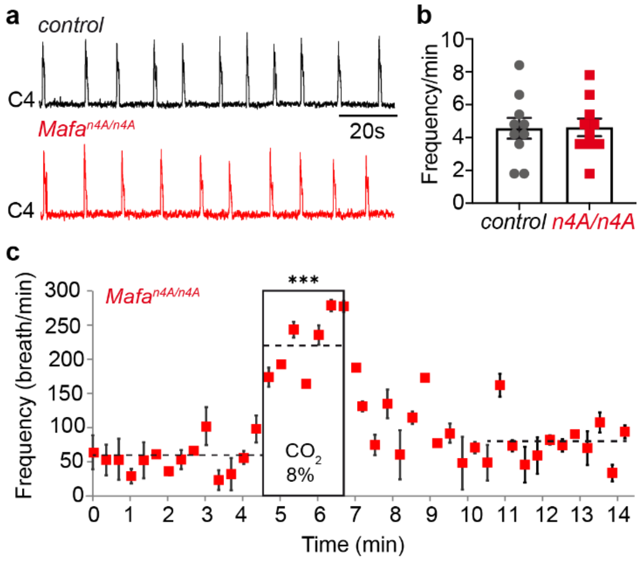

Supplementary Figure 4. The respiratory rhythm generator is spared in *Mafa*$^{n4A/n4A}$ mutants. a Integrated neurograms of the fourth cervical root (C_4) in isolated brainstem-spinal cord preparation from control (black trace) and *Mafa*$^{n4A/n4A}$ (red trace) P0 pups showing respiratory-like rhythmic activity. **b** Quantification histogram for control *(n=10)* and *Mafa* $^{n4A/n4}$ (n=11) preparations (two-sided unpaired Student's t-test P=0.9432, df=18, t=0.07). (**c**) Plot of the breathing frequency of *Mafa*$^{n4A/n4A}$ pups (n=4) at P0+12h recorded by plethysmography before, during and after a two-minute exposure to 8% CO_2 enriched air showing increased breathing frequency during the CO_2 challenge (3.6 fold increase; before/during CO2 treatment; two-sided unpaired Student's t-test P<0.0001, df=21, t=10.25) attesting of a preserved chemoceptive RTN. Horizontal dotted lines show average breathing frequencies before (60.5 $\pm$ 7.4 breath/min), during (219.3 $\pm$ 16.1 breath/min) and after (82.4 $\pm$ 9.5 breath/min) the CO_2 challenge. Symbols represent measurements (mean+/- sem) in 20s bins, from 4 animals.

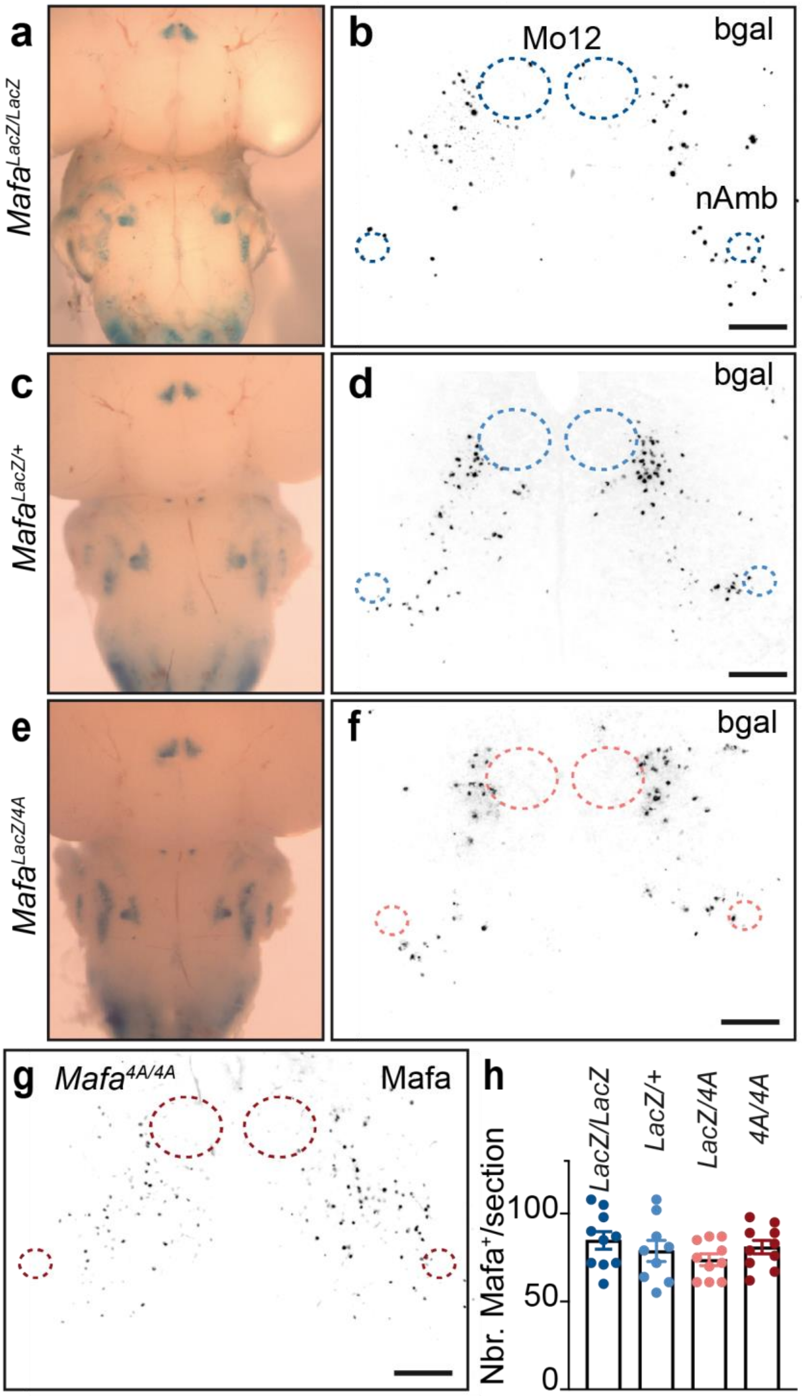

Supplementary Figure 5. *Mafa* mutations do not impair the development of Mafa$^+$ neurons. **a** Ventral view of a *Mafa*$^{LacZ/LacZ}$ brain wholemount at P0 (XGal staining, blue). Transverse section of the caudal medulla showing the distribution of Mafa$^+$ cells (bgal staining, black) in the reticular formation. **c**,**d** Same as **a**,**b** for *Mafa*$^{LacZ/+}$ mutant pup. **e**,**f** same as **a**,**b** for *Mafa*$^{LacZ/4A}$ mutant pup. **g** Distribution of Mafa$^+$ cells (Mafa immunostaining, black) in a *Mafa*$^{4A/4A}$ mutant pup. Note that the overall distribution of Mafa$^+$ neurons in the Peri12 and Peri-nAmb reticular formation is conserved across mutants, outlines of the Mo12 and nAmb are presumed. **h** Quantification (mean+/- sem) of Mafa$^+$ neurons in the caudal medulla reticular formation showing no significant differences across genotypes. Counts were made on

10 sections from 2 *Mafa*$^{LacZ/LacZ}$ pups, on 9 sections from 3 *Mafa*$^{LacZ/+}$ pups, on 10 sections from 2 *Mafa*$^{LacZ/4A}$ pups and on 10 sections from 2 *Mafa*$^{4A/4A}$ pups. Student's t-test comparing *Mafa*$^{LacZ/+}$ to other genotypes (*Mafa*$^{LacZ/LacZ}$: two-sided unpaired student t-tests P=0.45, df=17, t=0.77; *Mafa*$^{LacZ/4A}$: P=0.46, df=17, t=0.74; *Mafa*$^{4A/4A}$: P=0.75, df=17 t=0.3195). Scale bars (μm): 250 (**b,d,f,g,h**).

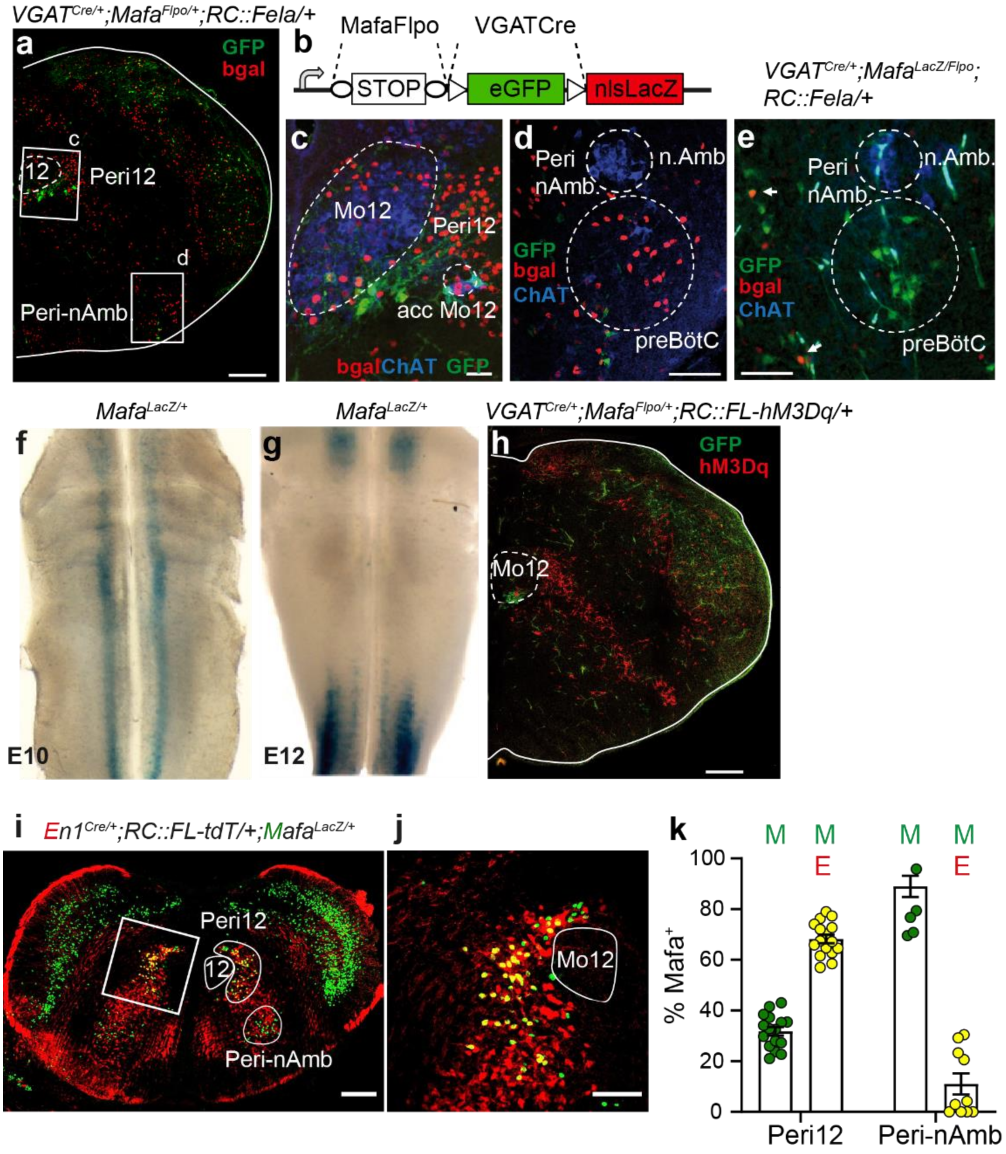

Supplementary Figure 6. PreBötC neurons transiently express Mafa at embryonic stage and Peri12 Mafa neurons are V1 type interneurons. **a** Medulla transverse hemi-section of an intersectional *VGAT*$^{Cre/+}$*;Mafa*$^{Flpo/+}$*RC::Fela/+* mutant pup at P0 showing the distributions of Mafa^{+}/VGAT^{+} inhibitory (bgal, red) and of Mafa^{+}/VGAT^{-} (GFP, green) neurons in the Peri12 and peri-nAmb region (repeated on n=3 *VGAT*$^{Cre/+}$*;Mafa*$^{Flpo/+}$*RC::Fela/+* pups). **b** Recombination scheme in the *R26*^{Fela} allele. **c** Close-up view of the c inset in (a) counterstained for ChAT (blue) showing Peri12 Mafa^{+} inhibitory neurons (red) and Mafa^{+}/ChAT^{+} motoneurons in the ventral Mo12 and its accessory nucleus (accMo12). **d** Close-up view of the d inset in (a) showing Mafa+ neurons with a history of expression of *VGAT* (red) in the Peri-nAmb. and in the preBötC. **e** Similar close-up view as d in a *Mafa*$^{LacZ/Flpo}$*;RC::Fela/+* mutant pup at P0 showing that preBötC neurons with a history of expression of Mafa (GFP) no longer express Mafa at P0 (absent red ßgal labeling) while neurons in the Peri-nAmb maintain Mafa expression (arrows). **f** Hindbrain flatmount (anterior at top) at E10 showing Mafa-expressing territories as continuous anterior-posterior stripes of expression on either side of the midline. **g** Same at E12 showing that Mafa expression has down regulated except in most anterior and posterior aspect of the rhombencephalon. **h** Medulla hemi-section of an intersectional

$VGAT^{Cre/+}$;$Mafa^{Flpo/+}$;*RC::hM3Dq*/+ mutant pup (repeated on n=2 $VGAT^{Cre/+}$;$Mafa^{Flpo/+}$;*RC::hM3Dq/+* pups) showing the distribution of hM3Dq-mCherry expressing (red) and Mafa^{+}/VGAT^{-} (GFP, green) neurons. **i** Transverse section of the caudal medulla of a $En1^{Cre/+}$;*RC::FL-tdT/+*;$Mafa^{LacZ/+}$ pup at P0 showing V1 type neurons (red), Mafa^{+} cells (green) and Mafa^{+} V1 type neurons (yellow). **j** Close-up view from the inset in (**a**) showing that more than half Mafa^{+} neurons (M) of the Peri12 area have a history of expression of En1 (ME) thus are V1 type neurons. **k** Quantification histogram showing that 67.8 $\pm$ 1.7% of all Mafa^{+} neurons in the Peri12 reticular formation (P.12) but only 10.7 $\pm$ 4.5% in the Peri-nAmb are V1 neurons (n= 3 pups). Scale bars (µm): 250 (**a**,**h**), 100 (**c**,**d**,**e**,**j**), 200 (**i**).

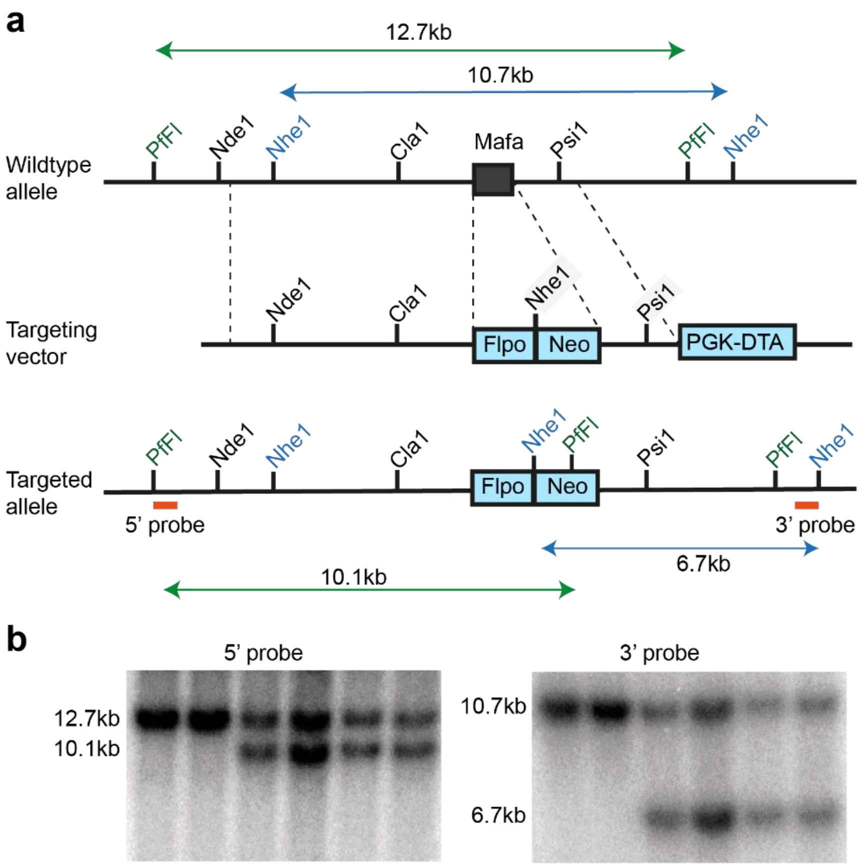

Supplementary Figure 7. Generation of *Mafa*$^{Flpo/+}$ mouse. a Strategy used to insert a Flpo and Neomycin selection cassette into the Mafa exon. **b** Recombination was confirmed by Southern blotting with an external 5' and 3'genomic probes. These constitutive *Mafa*^{Flpo} mutants were genotyped using Flpo specific primers.

Supplementary Table 1
Distribution of constitutive *Mafa* mutants at weaning (P21)

Crossing	*Mafa* $^{4A/+}$ x *Mafa* $^{LacZ/LacZ}$		*Mafa* $^{4A/+}$ x *Mafa* $^{4A/+}$		
Genotype	*Mafa*$^{LacZ/+}$	*Mafa* $^{LacZ/4A}$	*Mafa* $^{+/+}$	*Mafa* $^{4A/+}$	*Mafa* $^{4A/4A}$
Observed	15	16	73	89	0
Expected	16	16	73	146	73
χ^2	P=0.8981 χ^2 =0.01639 df=1		P<0.0001 χ^2 =54.03 df=2		
Survival	94%	100%	100%	60%	0%

Supplementary Table 2
Mafa mutants: Birth weight (g) and vocalization (P0+8h)

Genotype[a]	*Mafa*$^{+/+}$	*Mafa*$^{LacZ/LacZ}$	*Mafa* $^{4A/4A}$	n$^{cre/+}$; Mafa $^{flox4A/flox4A}$	n$^{+/+}$; Mafa$^{flox4A/flox4A}$
Weight	1.38 ± 0.03 n=13	1.28 ± 0.08 n=15	1.41 ± 0.05 n=10	1.23 ± 0.08 n=6	1.20 ± 0.06 n=8
	Mann-Whitney test two-sided	WT vs KO P=0.1657	WTvs4A4A P=0.8915	n$^{cre/+}$ vs n$^{+/+}$ P=0.966	
Vocalization (% time[b])	3.58±1.07 n=19	3.16±0.68 n=14	3.94±1.23 n=8	4.51± 1.25 n=14	3.21±1.27 n=13
	unpaired Student's t-test	WT/KO P=0.7074 t=0.3789	WT/4A4A P=0.8227 t=0.2281	n$^{cre/+}$ vs n$^{+/+}$ Mann-Whitney test P=0.0718 two-sided	

[a]*Mafa*$^{+/+}$, *Mafa* $^{LacZ/LacZ}$ and *Mafa* $^{4A/4A}$ were raised in 129Sv background whereas *nestin*$^{cre/+}$ (*n*$^{cre/+}$)*;Mafa* $^{flox4A/flox4}$ and *n*$^{+/+}$*;Mafa* $^{flox4A/flox4A}$ were raised in a mixt C57Bl6/jx129Sv background with lower body mass. [b] Measured in 5 minutes recordings.

Supplementary Table 3
Distribution of conditional *Mafa* mutants at weaning (P21)

Crossing	*nestin* $^{cre/+}$ *Mafa* $^{flox4A/+}$ *x nestin* $^{+/+}$ *Mafa* $^{flox4A/flox4A}$			
Genotype	$n^{+/+}$;*Mafa* $^{flox4A/+}$	$n^{cre/+}$;*Mafa* $^{flox4A/+}$	$n^{+/+}$;*Mafa* $^{flox4A/flox4A}$	$n^{cre/+}$;*Mafa* $^{flox4A/flox4}$
Observed	31	29	21	3
Expected	31	31	31	31
χ^2	P=0.0004 χ^2 =18.02 df=3			
Survival	100%	93%	67%	10%

Supplementary Table 4
Distribution of conditional *Mafa* mutants at birth (P0)

Crossing	*Mafa* $^{flox4A/+}$ *x Mafa* $^{flox4A/+}$		
Genotype	*Mafa* $^{+/+}$	*Mafa* $^{flox4A/+}$	*Mafa* $^{flox4A/flox4}$
Observed	18	34	23
Expected	18	37	18
χ^2	P=0.7013 χ^2 =0.7096 df=2		
Survival	100%	93%	+100%

Supplementary Table 5
Mouse strains

Strain	***Allele symbol***	***MGI: ID***
En1^{Cre}	Tg(Nes-cre)1Kln[1]	MGI:2176173
Mafa^{floxLacZ}	Mafa tm1 Eyc[2]	MGI:4868450
Mafa^{flox4a}	Mafa tm2 (4A) Eyc	MGI:6459714 (reserved allele)
Mafa^{flpo}	Mafa tm1 (Flpo) Gld	MGI:6459713 (reserved allele)
Nestin^{Cre}	Tg(Nes-cre)1Kln[3]	MGI:2176173
RC::FeLa	Gt(ROSA)26Sortm5(CAG-EGFP,-lacZ)Dym[4]	MGI:3795199
RC::FL-hM3Dq	Gt(ROSA)26Sortm3.2(CAG-EGFP,-CHRM3*/mCherry /Htr2a)Pje[5]	MGI:5771695
VGAT^{Cre}	Tg(Slc32a1-cre)#Oki[6]	MGI:6117419

References

1 Sapir, T. *et al.* Pax6 and engrailed 1 regulate two distinct aspects of renshaw cell development. *J Neurosci* **24**, 1255-1264, doi:10.1523/JNEUROSCI.3187-03.2004 (2004).
2 Lecoin, L. *et al.* MafA transcription factor identifies the early ret-expressing sensory neurons. *Dev Neurobiol* **70**, 485-497, doi:10.1002/dneu.20790 (2010).
3 Tronche, F. *et al.* Disruption of the glucocorticoid receptor gene in the nervous system results in reduced anxiety. *Nat Genet* **23**, 99-103 (1999).
4 Jensen, P. *et al.* Redefining the serotonergic system by genetic lineage. *Nature neuroscience* **11**, 417-419, doi:10.1038/nn2050 (2008).
5 Sciolino, N. R. *et al.* Recombinase-Dependent Mouse Lines for Chemogenetic Activation of Genetically Defined Cell Types. *Cell Rep* **15**, 2563-2573, doi:10.1016/j.celrep.2016.05.034 (2016).
6 Hagglund, M. *et al.* Optogenetic dissection reveals multiple rhythmogenic modules underlying locomotion. *Proceedings of the National Academy of Sciences of the United States of America* **110**, 11589-11594, doi:10.1073/pnas.1304365110 (2013).

Supplementary Table 6
Sequence of oligonucleotides used in qPCR experiments

Gene	***Species***	***Forward primer***	***Reverse primer***
MAFA	human	TTCAGCAAGGAGGAGGTCAT	GGCTCTGGAGTTGGCACTT
GAD1	human	CAAGTTCTGGCTGATGTGGA	CTCGCCATTGAAAACCATCT
GAD2	human	TGGCGATGGGATATTTTCTC	TCAGACGTGAAGGCAATGAG
Gad1	mouse	CACAAACTCAGCGGCATAGA	GGAAGAGGTAGCCTGCACAC
Gad2	mouse	CGCACTCTGGAAGACAATGA	ACCATGCGGAAGAAGTTGAC
TBP	mouse	ACATCTCAGCAACCCACACA	GGGTCATAGGAGTCATTGG
TBP	human	CACGAACCACGGCACTGATT	TTTTCTTGCTGCCAGTCTGGAC
