# Supplementary material for: Mafa-dependent GABAergic activity promotes mouse neonatal apneas
Source: Nat Commun. 2022 Jun 7;13:3284. doi: 10.1038/s41467-022-30825-3 (PMC9174494; doi:10.1038/s41467-022-30825-3)
Supplement: Supplementary file 3 — Reporting Summary [file 41467_2022_30825_MOESM3_ESM.pdf]

nature research

Corresponding author(s): Laure Lecoin, Celio Pouponnot and Gilles Fortin

Last updated by author(s): Apr 15, 2022

Reporting Summary

Nature Research wishes to improve the reproducibility of the work that we publish. This form provides structure for consistency and transparency in reporting. For further information on Nature Research policies, see Authors & Referees and the Editorial Policy Checklist .

Statistics

For all statistical analyses, confirm that the following items are present in the figure legend, table legend, main text, or Methods section.

n/a	Confirmed	
☐	☒	The exact sample size (*n*) for each experimental group/condition, given as a discrete number and unit of measurement
☐	☒	A statement on whether measurements were taken from distinct samples or whether the same sample was measured repeatedly
☐	☒	The statistical test(s) used AND whether they are one- or two-sided<br>*Only common tests should be described solely by name; describe more complex techniques in the Methods section.*
☒	☐	A description of all covariates tested
☒	☐	A description of any assumptions or corrections, such as tests of normality and adjustment for multiple comparisons
☐	☒	A full description of the statistical parameters including central tendency (e.g. means) or other basic estimates (e.g. regression coefficient) AND variation (e.g. standard deviation) or associated estimates of uncertainty (e.g. confidence intervals)
☐	☒	For null hypothesis testing, the test statistic (e.g. *F*, *t*, *r*) with confidence intervals, effect sizes, degrees of freedom and *P* value noted<br>*Give P values as exact values whenever suitable.*
☒	☐	For Bayesian analysis, information on the choice of priors and Markov chain Monte Carlo settings
☒	☐	For hierarchical and complex designs, identification of the appropriate level for tests and full reporting of outcomes
☒	☐	Estimates of effect sizes (e.g. Cohen's *d*, Pearson's *r*), indicating how they were calculated

Our web collection on statistics for biologists contains articles on many of the points above.

Software and code

Policy information about availability of computer code

Data collection	DNA strider 1.4f2 & Serial Cloner v2.6.1<br>Elphy (http://yzerlaut.github.io/Elphy/)<br>Avisoft recorder software v4.2.31<br>Spike2 v7<br>Leica Application Suite X v1.9.0.13747.1<br>NDPview2+ v2.6.17
Data analysis	Excell v14.7.7<br>Graph Pad Prism v9.3.1<br>Avisoft SASLab lite v5.2.15<br>Image J v2.0.0-rc-69/1.52p<br>Adobe photoshop CS6 v16.0.3

For manuscripts utilizing custom algorithms or software that are central to the research but not yet described in published literature, software must be made available to editors/reviewers. We strongly encourage code deposition in a community repository (e.g. GitHub). See the Nature Research guidelines for submitting code & software for further information.

Data

Policy information about availability of data

All manuscripts must include a data availability statement. This statement should provide the following information, where applicable:

- Accession codes, unique identifiers, or web links for publicly available datasets
- A list of figures that have associated raw data
- A description of any restrictions on data availability

The data that support the findings of this study can be found in the Source Data provided with the paper. Microscopy data are available from the corresponding authors upon reasonable request.

Field-specific reporting

Please select the one below that is the best fit for your research. If you are not sure, read the appropriate sections before making your selection.

☒ Life sciences ☐ Behavioural & social sciences ☐ Ecological, evolutionary & environmental sciences

For a reference copy of the document with all sections, see nature.com/documents/nr-reporting-summary-flat.pdf

Life sciences study design

All studies must disclose on these points even when the disclosure is negative.

Sample size	The sample sizes used in this study are stated in the legends of relevant figures and in the "statistics and reproducibility" statement, and are consistent with those of comparable anatomical and functional studies in the field (e.g. doi:10.1016/j.neuron.2012.11.010., 10.1016/j.neuron.2010.10.019., 10.1523/JNEUROSCI.1721-11.2011., 10.1038/s41586-020-2991-4). Sample sizes were chosen to support meaningful conclusions in accordance with ethical committee requirements to limit the use of animals, whilst being adequate in magnitude, to ensure the consistency of qualitative and quantitative observations.
Data exclusions	There are no data exclusions.
Replication	Respiratory/vocalization behavior experiments were replicated on individual littermates from several litters and for respiratory behavior across multiple time points per individual. All anatomical experiments were reproduced a minimum of three times. The n and variability (reported as SEM) are given in text and figure legends.
Randomization	Randomization is not applicable in any of our studies:<br>-In respiratory and vocalization experiments, all pups (mutants and controls to ensure genetic homgeneity) were systematically recorded. Due to the small size of litters, the low frequency of mutants (typically 1/8 pups) and their short life, no randomization could be performed.<br>-In descriptive anatomical experiments, randomization does not apply as there were no group allocation. We made qualitative and quantitative assessments of the data across multiple individuals of the same type for each experimental series.<br>-In comparative anatomical experiments, group allocation corresponded to a genotype and the sample sizes were chosen to support meaningful conclusions in accordance with ethical committee requirements to limit the use of animals, whilst being adequate in magnitude, to ensure the consistency of qualitative and quantitative observations.
Blinding	Blinding for functional data collection was imposed by systematic post-experimental genotyping of the recorded individuals. Blinding was not used for data analysis which was done in parallel by distinct experimenters.

Reporting for specific materials, systems and methods

We require information from authors about some types of materials, experimental systems and methods used in many studies. Here, indicate whether each material, system or method listed is relevant to your study. If you are not sure if a list item applies to your research, read the appropriate section before selecting a response.

Materials & experimental systems

n/a	Involved in the study	
☐	☒	Antibodies
☐	☒	Eukaryotic cell lines
☒	☐	Palaeontology
☐	☒	Animals and other organisms
☒	☐	Human research participants
☒	☐	Clinical data

Methods

n/a	Involved in the study	
☒	☐	ChIP-seq
☒	☐	Flow cytometry
☒	☐	MRI-based neuroimaging

Antibodies

Antibodies used

Primary antibodies:
-rabbit anti-Mafa (Bethyl, IHC-00352, 1/200)
-chicken anti-ßGal (Abcam, ab9361, 1/1000)
-goat anti-ChAT (Millipore, AB 144P, 1/1000)
-rabbit anti-RFP (Rockland, 600-401-379, 1/500)
-chicken anti-GFP (Aves, GFP1020, 1/1000)
-rabbit anti-NK1R (Sigma, S8305, 1/5000).

Secondary antibodies:
All secondary antibodies were used at 1:500 dilution.
-donkey anti-rabbit Cy5 (Jackson laboratories, 712-165-153)
-donkey anti-chicken 488 (Jackson laboratories, 703-545-155)
-donkey anti-chicken Cy5 (Jackson laboratories, 703-176155)
-goat anti-Rabbit Cy3 (Invitrogen, A10520)
-donkey anti-goat Cy5 (Jackson laboratories, 705-606-147)

Validation

-Rabbit anti-Mafa: Species reactivity for mouse as per the manufacturer the validation standardized process: https://www.fortislife.com/antibody-validation also validated by DOI: 10.1038/s41467-020-20632-z.

-Chicken anti-ßGal: Species reactivity for mouse as per the manufacturer, P0-adult mice were euthanized and perfused with 4% paraformaldehyde in PBS (PF). Their spinal cords were then post-fixed for 30–60 mins in 4% PF at 4°C (P0) or at room temperature(adult). Spinal cords were rinsed and cryoprotected in 20% sucrose in PBS (4°C) prior to embedding in OCT (Tissue-Tek). Immunostaining of frozen spinal sections was performed by incubating 20 µm thick sections with primary antibodies, which were then detected using species-specific secondary antibodies conjugated with Cy2, Cy3 and Cy5 or FITC. ab9361 was used at 1:1000. doi: 10.1371/journal.pone.0077928

-Goat anti-ChAT: Species reactivity for human, rat, mouse, monkey, opossum, avian, chicken, guinea pig, zebrafish as per the manufacturer, "Goat anti-ChAT (Catalogue No. AB144P) staining of organotypic slice cultures of septum from 7-day-old rat tissue maintained in culture for 8 days (IHC). Coronal section of rat neocortex stained with catalog number AB144P showing the choline acetyltransferase positive cholinergic nerve fibers and terminals. Reference: D. Anandh, K Shobha and Dr. Bindu M Kutty, Department of Neurophysiology, National Institute of Mental Health and Neurosciences, Bangalore, India.Western Blot Analysis: Representative lot data. NIH/3T3 lysate was resolved by electrophoresis, transferred to PVDF membrane and probed with anti-CHAT (1:1000 dilution). Proteins were visualized using a rabbit anti-goat secondary antibody conjugated to HRP and a chemoluminescence detection system. Validated via IHC in Mouse by doi: 10.7554/eLife.06412.

-Rabbit anti-RFP: Species reativity for human, mouse, rat as per the manufacturer, Polyclonal anti-RFP is designed to detect RFP and its variants. This antibody has been used in WB, IP, IF, ICC, IHC, dual RNA-FISH, iDISCO+, IEM, and FLOW. This antibody can be used to detect RFP by ELISA (sandwich or capture) for the direct binding of antigen. This product was prepared from monospecific antiserum by immunoaffinity chromatography using Red Fluorescent Protein (Discosoma) coupled to agarose beads followed by solid phase adsorption(s) to remove any unwanted reactivities. Expect reactivity against RFP and its variants: mCherry, tdTomato, mBanana, mOrange, mPlum, mOrange and mStrawberry. Assay by immunoelectrophoresis resulted in a single precipitin arc against anti-Rabbit Serum and purified and partially purified Red Fluorescent Protein (Discosoma). No reaction was observed against Human, Mouse or Rat serum proteins. Used in doi: 10.1016/j.devcel.2021.12.012.

-Chicken anti-GFP: Species reactivity for mouse as per manufacturer, "Antibodies were analyzed by western blot analysis (1:5000 dilution) and immunohistochemistry (1:500 dilution) using transgenic mice expressing the GFP gene product. Western blots were performed using BlokHen® (Aves Labs) as the blocking reagent, and HRP-labeled goat anti-chicken antibodies (Aves Labs, Cat. #H-1004) as the detection reagent. Immunohistochemistry used tetramethyl rhodamine-labeled anti-chicken IgY." Validated via IHC in Mouse by doi:10.1523/ENEUR0.0174-16.2016

-Rabbit anti-NK1R: Species reactivity for human, rat, guinea pig, mouse as per manufacturer, Anti-Substance P Receptor (NK1R, SPR) reacts specifically with NK1R (46 kDa), derived from rat brain. The antibody may be used in immunoblotting of rat brain membrane fraction extracts and in immunohistochemical staining of 4%paraformaldehyde/0.2% picric acid/0.05% glutaraldehyde perfusion-fixed frozen tissue sections of rat brain. Staining of the NK1R band (46 kDa) in immunoblotting is specifically inhibited with NK1R peptide (rat, amino acids 393-407). doi: 10.1152/physrev.1993.73.2.229.

Eukaryotic cell lines

Policy information about cell lines

Cell line source(s)

DAOY and HEK293T cell lines were purchased from ATCC.

Authentication	Cell lines are regularly authenticated by STR (short tandem repeat) testing.
Mycoplasma contamination	Cell lines were tested negative for mycoplasma contamination.
Commonly misidentified lines (See ICLAC register)	No commonly misidentified cell lines were used in this study.

Animals and other organisms

Policy information about studies involving animals; ARRIVE guidelines recommended for reporting animal research

Laboratory animals	Mus musculus, strain C57Bl6/J, neonatal pups aged postnatal day (P) P0 to P9 of either sex. The unpublished mouse knock-in: Mafa tm2(4A) Eyc and Mafa tm1 (Flpo) Gld have been submitted to MGI database (respective accession numbers: MGI:6459714 and MGI:6459713). Other mouse strains described in this study (Mafa tm1 Eyc; Tg(Nes-cre)1Kln; Slc32a1tm2(cre)low; En1tm2 (cre)Gld; Gt(ROSA)26Sortm5(CAG-EGFP,lacZ)Dym; Gt(ROSA)26Sortm3.2(CAG-EGFP,-CHRM3*/mCherry/Htr2a)Pjen have already been reported.
Wild animals	No wild animals were used in this study.
Field-collected samples	No field collected samples were used in this study.
Ethics oversight	All experimental procedures and the handling of mice were done in accordance with the European Community Directive 86/609/EEC and following the recommendations of the French National Ethics Committee for Science and Health report on “Ethical Principles for Animal Experimentation” - CEEA n°59 Paris Centre et Sud – under agreement N° 2015071710462096.

Note that full information on the approval of the study protocol must also be provided in the manuscript.
